# The Increasing Issue of Vancomycin-Resistant Enterococci and the Bacteriocin Solution

**DOI:** 10.1007/s12602-019-09618-6

**Published:** 2019-11-22

**Authors:** Ingvild S. Reinseth, Kirill V. Ovchinnikov, Hanne H. Tønnesen, Harald Carlsen, Dzung B. Diep

**Affiliations:** 1 Department of Chemistry, Biotechnology and Food Science, Norwegian University of Life Sciences, P.O. Box 5003, 1432 Ås, Norway; 2 Section of Pharmaceutics and Social Pharmacy, Department of Pharmacy, University of Oslo, P.O. Box 1068 Blindern, 0316 Oslo, Norway

**Keywords:** *Enterococcus faecium*, *Enterococcus faecalis*, VRE, Antibiotic resistance, Virulence, Bacteriocin

## Abstract

Enterococci are commensals of human and other animals’ gastrointestinal tracts. Only making up a small part of the microbiota, they have not played a significant role in research, until the 1980s. Although the exact year is variable according to different geographical areas, this was the decade when vancomycin-resistant enterococci (VRE) were discovered and since then their role as causative agents of human infections has increased. *Enterococcus faecium* is on the WHO’s list of “bacteria for which new antibiotics are urgently needed,” and with no new antibiotics in development, the situation is desperate. In this review, different aspects of VRE are outlined, including the mortality caused by VRE, antibiotic resistance profiles, animal-modeling efforts, and virulence. In addition, the limitations of current antibiotic treatments for VRE and prospective new treatments, such as bacteriocins, are reviewed.

## Introduction

Vancomycin-resistant enterococci (VRE) emerged from the commensal enterococci in the 1980s and have developed from “generally regarded as safe” bacteria to significant nosocomial pathogens [2, 3, 21, 23, 45, 53, 68, 93, 109]. The World Health Organization (WHO) still considers VRE a pathogen with high priority on its global priority list [116]. VRE exist in the gastrointestinal tract of healthy humans and other animals, but may colonize and disseminate if conditions are suitable [1, 15, 57, 71, 97, 111]. Suitable conditions for colonization include treatment with anti-anaerobic antibiotics including vancomycin, which remove colonization resistance and provide a vacant niche for the VRE to invade [15, 84, 116]. In this context, colonization is the establishment of vancomycin-resistant enterococcal populations in the gastrointestinal tract due to displacement of the non-resistant enterococcal counterparts. Dissemination is the spread from the gastrointestinal tract and thereby start of an infection. Enterococci are innately resistant to many classes of antibiotics, but when they acquire additional resistance through, for example, mobile genetic elements, they become increasingly difficult to treat [54, 67, 101]. The factors that contribute to virulence in VRE are not completely characterized, but factors such as enterococcal surface protein, aggregation substance, gelatinase, and collagen adhesin molecule Acm have been implicated in the ability to colonize different tissues [23, 41, 60, 68, 70]. VRE can colonize the gastrointestinal tract of mice after antibiotic treatment and additional treatment with an immunocompromising agent allows dissemination in murine models [40, 79, 120]. Physicians have limited treatment options and resistance to new antibiotics is rising. There is now a pressing need for new classes of antibiotics that can deal with antibiotic resistant pathogens. Small antimicrobial peptides, bacteriocins, may be a new treatment alternative, either as the only treatment or in synergy with existing antimicrobial compounds [27, 114]. In this article, we will review these aspects in light of the significant threat VRE contribute to the era of antibiotic resistance, with special focus on factors facilitating the dissemination of VRE across the gastrointestinal tract to cause systemic infection, our current drug arsenal against VRE infections, and the potential of bacteriocins as alternative drugs and/or supplements in VRE treatments.

## VRE Cause Significant Mortality

VRE are increasingly becoming a larger problem for hospitals worldwide, and due to VRE’s capabilities to survive for longer periods on inanimate surfaces (such as benches, beds, implanted surgical devices, and ventilation systems) and role as a commensal, it is increasingly difficult to control their spread [4, 90]. VRE cause a range of infections, from bacteremia, endocarditis, urinary tract infections, intra-abdominal, and pelvic infections, to peritonitis, skin infections, skin-structure infections, and central nervous system infections [29, 84, 90, 116, 128]. The attributable mortality of VRE infections is difficult to determine due to the common contribution of underlying disease, i.e., that the patient often is suffering from another condition prior to infection [104]. Examples of these conditions are cancerous conditions and transplant patients. The pathway of a VRE infection often starts with gastrointestinal colonization due to loss of colonization resistance through destabilization of the gut microbiota [39, 90]. Therefore, risk factors associated with promoting VRE infection include antibiotic exposure [105], comorbid illness [90], and patients that are immunocompromised, like patients undergoing chemotherapy [66]. In addition, factors such as prolonged hospital stay or an indwelling device, such as a catheter, breach a barrier and facilitate a proximity that gives the enterococci opportunity to cause infection [4, 82, 123]. A study among Pakistani hospitalized cancer patients indicated a 12-week mortality rate of 63% for VRE-bacteremia patients [4]. However, earlier studies have found lower mortality rates, for example, a study from several hospitals performed on patients with established VRE infection reported a 19% mortality rate, while a case-control matched study found an VRE attributable mortality of 37% [42, 123]. These studies report VRE mortality between 19% and 63%, and were performed on limited samplings, such as a few hospitals in one geographical area, and have a variability in the patient cohort included. Therefore, a future prospect should be to undertake investigations of a larger area, large cohort and continue to case-control match the patients, in order to gain a full understanding of the mortality burden caused by VRE.

Previous reports have indicated that VRE cause more mortality than infections caused by vancomycin-susceptible enterococci (VSE) [4, 94, 110]. This indicates either that some aspects of the resistant infections are more pathogenic than infections caused by the susceptible pathogen or it may reflect the timespan until effective treatment of the infection is reached. However, two factors may confound the results. Firstly, *E. faecium* has a higher rate of vancomycin resistance than *E. faecalis,* and the majority of VRE infections are caused by *E. faecium*, giving a skewed sampling. Secondly, there is more comorbidity in VRE infections than VSE, which makes determining cause of morbidity difficult [94].

## Enterococci and Antibiotic Resistance

The vancomycin-resistant enterococci are facultative anaerobic, oval, Gram-positive cocci with high innate antibiotic resistance as well as significant acquired antibiotic resistance [8]. Intrinsic and acquired resistance differ in that, in general, genes that are present on the chromosome encode intrinsic resistance, while mobile genetic elements, such as plasmids and transposons, encode for acquired resistance [101]. Mobile genetic elements have been shown to play an important role in the acquired resistance of *Enterococcus* spp., and this has been reviewed elsewhere [54]. Enterococci intrinsically carry resistance to penicillin, aminoglycosides, clindamycin, and cephalosporins. In addition, they may require resistance to other β-lactams and glycopeptide antibiotics such as vancomycin [73, 82]. The different resistance mechanisms of enterococci have been reviewed elsewhere [67]. The most common nosocomial enterococci are *E. faecalis* and *E. faecium.* Previously *E. faecalis* was the most frequently isolated strain of the two, but a shift seems to have caused *E. faecium* to prevail in recent years [69, 111]. Interestingly, in most cases, *E. faecium* has a more extensive antibiotic resistance profile than *E. faecalis*, even though the latter has intrinsic resistance to the streptogramin quinupristin/dalfopristin and is hypothesized as the more virulent of the two [23, 67].

There are surveillance programs in place for monitoring incidences of VRE in both Europe, and other continents. Recent reports indicate an increasing trend of vancomycin resistance among *E. faecium* in Europe. Northern Europe continues to have a low percentage, but significant travel across European borders likely facilitates the spread of all resistant bacteria. The regional director of the World Health Organization stated that “antimicrobial resistance is increasingly widespread in the WHO European Region as resistant microbes know no borders” [116].

Different *van*-plasmids carry several genes necessary for vancomycin resistance. These genes encode enzymes that not only produce the alternative cell wall precursor D-Ala-D-Lac or D-Ala-D-Ser, depending on the type of *van*-plasmid, but also prevent synthesis of the original D-Ala-D-Ala [82]. Carrying the vancomycin resistance trait does not lower fitness, indicating that there is some type of regulatory system to induce the *van* genes when vancomycin is present [67]. The two most commonly found plasmids are the *vanA* and *vanB* plasmids, with *vanA* conferring the highest resistance [84]. Currently, nine different *van*-operons conferring different levels of vancomycin resistance are characterized [67, 84]. Vancomycin is a last resort antibiotic, an antibiotic used when other treatments fail due to resistance. It is therefore worrisome that resistance toward this last resort antibiotic spreads through an opportunistic species such as *E. faecium* and likely results in a relevant clinical impact [124].

## Nosocomial Strains of VRE Are Enriched in Putative Virulence Factors

Several studies have investigated the importance of putative virulence genes in VRE, but there are no clear conclusions as to what constituents decisively contribute to the pathogenicity of VRE yet. Although it is difficult to prove the correlation between a virulence factor and the ability of a strain to cause infection, the general trend observed is that nosocomial strains carry the gene in question while indigenous strains do not. The only virulence genes confirmed to be associated with VRE infection are the enterococcal surface protein gene (*esp*) and the hyaluronidase gene (*hyl*) [69, 70]. The putative virulence factors include proteins that attack several different constituents of cells, such as cytolysin that targets cell membranes, as well as gelatinase and serine protease that attack various proteins such as collagen, fibrinogen, and insulin. In addition, binding proteins such as collagen-binding protein, enterococcal surface protein, and aggregation substance are putative virulence factors [41, 60, 68, 71]. Furthermore, it has been suggested that the different factors may have different roles in the different resistant *Enterococcus* species. One example of this is the frequently discussed virulence factor, enterococcal surface protein, Esp. It has been shown that Esp plays a vital role in the establishment of vancomycin resistant *E. faecium* urinary tract infection in animal models, but the role of Esp in *E. faecalis* is not clear. Therefore, it is possible to speculate that the Esp protein plays a less important role in *E. faecalis*, which is supported by the finding of Esp in both indigenous and nosocomial strains of *E. faecalis*, while the distribution of Esp in *E. faecium* is mostly limited to nosocomial strains [70]. One study investigating putative virulence factors in enterococci isolated from both the environment, food and nosocomial strains, further supported the differences between *E. faecalis* and *E. faecium* [71]. The virulence factors present in *E. faecalis* varied between strains, and the different strains contained combinations of cytolysin, gelatinase, and surface protein. *E. faecium* strains only contained surface protein, and no other putative virulence determinants [71]. Another virulence factor involved in adhesion is Acm, which is a member of the microbial surface components recognizing adhesive matrix molecules, MSCRAMMs. This protein is necessary for the bacteria to bind to collagen type 1 in vancomycin-resistant *E. faecium* isolates. Considering that adhesion to constituents of the extracellular matrix is necessary for the bacteria to colonize and invade tissues, it may be classified as a virulence factor [83].

A connection between a strain carrying virulence traits and a strain being vancomycin resistant has been evaluated with different conclusions. One study found that when investigating clinical strains, there was no correlation between a strain having virulence traits, and a strain being vancomycin resistant [23]. However, other studies have found that such correlation does exist [101], and even indicated that some plasmids carrying resistance genes also carry virulence factors [89].

## Key Factors for VRE Infection and Dissemination

The mouse model is a well-established system to study the effect of infection, and this includes infections caused by VRE. Studies have investigated the processes that occur before and during the colonization and possible dissemination of VRE in mice. These studies have considerably increased our understanding of the interaction of VRE with the gut microbiota and the effect of antibiotic administration. Several studies indicate that the effect of antibiotic administration is profound on the composition of the microbiota, the bacteria associated with the human gastrointestinal tract, as well as on the ability of VRE to colonize [12, 40, 79, 115, 120].

The general tendency observed in controlled in vivo experiments, is that antimicrobials targeting anaerobic bacteria, for example metronidazole and β-lactams, promote VRE colonization, while antibiotics with less anti-anaerobic effect have less effect on the ability of VRE to colonize the gastrointestinal tract [40, 115]. In general, the density and diversity of the gastrointestinal microbiota decrease during antibiotic treatments, and although the density increases when the antibiotic treatment is discontinued, the composition is altered. Specifically, the populations of the phyla *Lactobacillaceae* and *Bacteroides* are distinctly decreased and the post-antibiotic microbiota consists of a larger number of *Clostridium* and *Enterococcus* species [120]. Antibiotics that reduce the population of *Enterobacteriaceae* and *Lactobacillus* spp. include kanamycin, ampicillin, metronidazole, neomycin, and vancomycin [79, 120]. Several antimicrobial treatments have an increasing effect on *Enterobacteriaceae* and *Enterococcus* spp., most likely due to eradication of competition. This includes metronidazole, vancomycin, and neomycin. The consequence is that administration of these drugs to patients increases the amount of enterococci in the gut, thereby increasing the possibility of dissemination and infection. The administration of an immune suppressant in the absence of antibiotic treatment does not influence the normal intestinal flora, indicating that change in microbiota is not the reason for the increased susceptibility of enterococcal infection in immunocompromised patients [79].

Different antimicrobials have distinct effects on the ability of VRE to colonize the gastrointestinal tract, through their interaction with the microbiota and the inability of the antibiotics to target VRE. Treatment of mice with different combinations of metronidazole, neomycin, kanamycin, and vancomycin increased colonization with VRE in several studies [12, 79, 120]. Anti-anaerobic antibiotics such as piperacillin, tazobactam, cefoxitin, and clindamycin promoted VRE colonization in a mouse model. In the same model, cefepime and aztreonam, which are antibiotics with little anti-anaerobic activity, did not facilitate VRE colonization. The combined antibiotic piperacillin/tazobactam is a drug with anti-enterococcal effect, but it also has potent anti-anaerobic effect. Studies showed that during treatment, VRE colonization was prevented. However, since the anaerobic microbiota was also targeted, VRE was able to colonize after discontinuation of treatment. This indicates that piperacillin/tazobactam is able to prevent VRE colonization to some degree, but that VRE persists in the gastrointestinal tract and is able to re-colonize upon discontinuation of the antibiotic treatment [40, 115].

Colonization is critical in the process of VRE infection. It has been shown that the commensal microbiota influences the ability of opportunistic pathogens to colonize in both a direct and an indirect route. The direct route is that the commensal bacteria occupy the niches that opportunistic species need to colonize, and they are better adapted, so that they displace the opportunists, a process referred to as competitive exclusion [15]. In addition, the commensal bacteria have been shown to interact with the innate immune system of the host, stimulating production of antimicrobial compounds, such as the C-type lectin RegIIIγ [12]. These antimicrobial compounds have minimal effect toward the commensals themselves, but have potent antimicrobial activity toward several Gram-positive opportunists, including the VRE [13].

A few studies have measured VRE dissemination after VRE has colonized the gut, a process believed to be necessary for the ability of VRE to cause systemic infection [12, 55, 79]. Studies have shown that administering a broad-spectrum antibiotic mixture consisting of neomycin, metronidazole, and vancomycin to mice followed by oral inoculation with VRE is sufficient to induce VRE colonization of the gastrointestinal tract and dissemination to other tissues. One study found that broad-spectrum antibiotic treatment and subsequent VRE inoculation were sufficient for VRE to disseminate to the blood of the treated animals [12]. Another study also investigated whether VRE had spread to tissues such as the liver and spleen [79]. In this study, the mice were administered cyclophosphamide in addition to the antibiotic mixture, and therefore were immunocompromised in addition to having their microbiota altered. They discovered VRE dissemination to mesenteric lymph nodes, liver, spleen as well as blood. Whether immunocompromising treatment is necessary for dissemination to tissues is unclear. Retrospective studies in humans provide support for the notion that the individual needs to be immunocompromised in order for the dissemination to occur. For example, one study found that of 216 human patients colonized with VRE, only three developed bloodstream infections, and all patients that developed bloodstream infections were immunocompromised. It might be that immunosuppression is necessary for the dissemination of VRE in humans as well as in experimental animals [66].

## Is Esp Involved in Adherence?

Bacteremia with VRE may be dependent on the ability of the bacteria to colonize and translocate the intestinal tract [55]. This would depend on the ability of the bacteria to adhere to and invade the mucosal layer of the intestinal tract. One study in mice showed that the treatment with antibiotics, such as ampicillin, decreased the thickness of the mucus in the mucosal membrane. Colonization with *Klebsiella pneumoniae* restored the thickness of the colon mucus, but colonization with VRE did not. However, no invasion of the mucosal membrane was found in the antibiotic-treated, VRE-colonized mice [16], in line with previous notion that immunosuppression is necessary in combination with antibiotics for VRE dissemination. Some murine models have investigated the properties of the enterococci that facilitate their ability for high-level gastrointestinal colonization. The enterococcal surface protein (Esp) has been suggested as a factor that might be involved, especially since the encoding gene was enriched in clinical strains [101]. However, studies in mice did not indicate any difference in the colonization properties of neither *E. faecalis* nor *E. faecium* whether they had the *esp* gene or not [56, 95]. Furthermore, no difference was found in adherence to a human cell line in vitro, between an *esp-*positive *E. faecium* blood culture isolate, and the same strain with *esp* deleted [56]. This indicates that *esp* is not the factor responsible for adherence to human cell lines. In the same study, a significant difference in adherence was shown between a community-acquired *esp*-negative strain, and the blood culture *esp*-positive isolate. If one postulates that *esp* is not responsible for adherence, as indicated by the similar adherence in the same strain with and without *esp*, it suggests that the community isolates lack some other unknown adherence factors that the nosocomial blood isolates possess [56]. A study has shown that clinical strains of vancomycin-resistant *E. faecium* have the ability to bind to human intestinal mucus in vitro, but with lower affinity than other commensals of the gastrointestinal tract, indicating that the binding affinity of the different genera may play a part in the colonization resistance of the microbiota [96].

When VRE are able to cross over the gastrointestinal barrier and disseminate, other bacterial species may also spread. Miyazaki et al. found *E. coli* in the same blood samples as VRE, although outnumbered 100:1, indicating that both have followed the same infection route [79]. This correlates well with the suggestion that VRE might need the presence of other bacteria to cross over the mucus layer of the gastrointestinal tract, considering its limited ability to invade the mucus layer when mono-colonized [16].

## Though Effective Treatment Exists, VRE Continues to Cause Significant Mortality and Resistance Arises

VRE are commensals of the gastrointestinal tract, but do not normally colonize this organ [57, 68]. However, colonization of the gastrointestinal tract is believed to be the first step of infection, and therefore it is of great importance [12, 55, 71, 79, 97, 111]. Severe infection with VRE does require treatment, but due to the high antibiotic resistance, and the innate ability of enterococci to develop resistance toward new compounds quickly, there are few effective therapies available [78]. Currently considered effective are quinupristin-dalfopristin, tigecycline, teicoplanin, telavancin, linezolid, and daptomycin (Table 1) [94]. Some of these compounds are only approved as treatment for skin-related infections, and some are in the experimental phases of development (see Table 1). However, resistance to these new antimicrobials has been documented as early as 2001 for quinupristin-dalfopristin, and in fact, none of the new antibiotics are free from enterococcal resistance [67, 123].

**Table 1:** Current antimicrobials for VRE treatment and their mode of action

Antimicrobial	Mode of action	Reference
Quinupristin/dalfopristin	Protein translation; targets the 50S ribosomal subunit	[6]
Linezolid	Protein translation; targets the 50S ribosomal subunit	[85]
Tigecycline	Protein translation; targets the 30S ribosomal subunit blocking the entry of transfer RNA	[39]
Teicoplanin/telavancin	Cell wall; binds to cell wall precursor D-Ala-D-Ala preventing cross-linking	[53]
Daptomycin	Cell membrane; likely inserts in membrane in a calcium-dependent manner and causes leakage with subsequent depolarization	[53]
Tedizolid	Protein translation; targets the 23S ribosomal subunit	[90]
Oritavancin	Cell wall; binds to D-Ala-D-Ala of cell wall precursor preventing cross-linking; depolarization	[67]
Nisin	Associates with membrane lipid II and causes leakage with subsequent depolarization; inhibit cell wall synthesis	[64]
Garvicin KS	Unknown receptor; affects stress response	[71]
EF478	Serine protease-like structure, unknown mechanism	[77]
Enterocin K1	Associates with membrane RseP and causes leakage with subsequent depolarization	[72]
Enterocin EJ97	Associates with membrane RseP and causes leakage with subsequent depolarization	[72]

### Quinupristin-Dalfopristin—Streptogramin Antibiotics

Quinupristin-dalfopristin is a combined antibiotic composed of two synergistically acting constituents that both bind to the 50S ribosomal subunit and interfere with protein translation [7, 67, 72]. It is commonly used against infections with VRE, but several studies have found that resistance is emerging [72, 73, 98]. For example, Maraki et al. found that 17.1% of isolated nosocomial strains were resistant to quinupristin-dalfopristin [73]. The susceptibility to quinupristin-dalfopristin has been determined to depend on the species of VRE. While most *E. faecalis* strains are resistant to quinupristin-dalfopristin, the antibiotic does have substantial activity toward *E. faecium* [36, 72]. This difference in susceptibility is likely due to the *lsa* gene, encoding a putative ABC-transporter that transports the antibiotic away from its 50S rRNA target [67, 127]. *E. faecalis* therefore has intrinsic resistance to quinupristin-dalfopristin while *E. faecium* carries acquired resistance due to acetyltransferases that modify the rRNA target, and by genes that encode ABC transporters to efflux the antibiotic [67, 127]. In addition to the occurrence of resistance, treatment with quinupristin-dalfopristin involves side effects such as joint and muscle pain [127].

### Linezolid Has Bacteriostatic Effect

Linezolid is used for infections caused by antibiotic resistant Gram-positive organisms and has bacteriostatic effect by targeting the 23S rRNA subunit of the translational machinery [103]. It is reported by surveillance programs that the occurrence of resistance is rare, 1.83% resistance was reported in 2012 [37, 77]. Several studies have investigated the effect of linezolid on enterococcal infections. One large study found that among 138 patients that received linezolid treatment, there was approximately 18% overall mortality [119]. Two other studies reported higher mortality rates, 20.6% and 29.4%, with investigations in smaller patient cohorts, 68 and 34 patients, respectively [28, 74]. Resistance develops with some difficulty due to the fitness loss associated with an altered ribosomal subunit, resulting in less efficient protein translation [76]. Resistance development is normally associated with prolonged use of linezolid or invasive procedures [76]. Several resistance mechanisms have emerged in different types of antibiotic resistant bacteria, including VRE [34, 75, 76]. These resistance mechanisms include chromosomal modifications of the 23S rRNA subunit as well as the L3 and L4 accessory proteins [76]. Resistance may also be caused by the *cfr* gene which codes for an enzyme belonging to the radical S-adenosyl-L-methionine superfamily. Cfr methylates a carbon atom on the alanine in position 2503 in the 23S rRNA, protecting it from linezolid [34, 127]. It is possible that some strains that are non-susceptible to linezolid, but do not carry modifications in the 23S rRNA, L3 or L4, and do not carry the *cfr* gene, may contain efflux pumps that recognize linezolid, but this has not been proven yet [76]. The *cfr* gene is plasmid-located and associated with transposons and other mobile genetic elements; therefore, it is associated with a risk of dissemination [76].

### Tigecycline Is a Tetracycline Derivative

Tigecycline is a glycylcycline antibiotic, which is a class of antibiotics derived from tetracycline. It targets the 30S ribosomal subunit and blocks the transfer RNA, hindering protein translation. Tigecycline has an increased affinity for the 30S subunit, hence being more potent compared with tetracycline [50]. Tigecycline monotherapy is not recommended as it has been indicated that the antibiotic cannot achieve high enough serum concentration to achieve sufficient antibacterial effect [7]. The side effects caused by tigecycline can be significant, and in combination with the low serum concentration, it may have limited value as a VRE therapy [127].

### Teicoplanin and Telavancin Are Lipoglycopeptides

Teicoplanin and telavancin are semisynthetic derivatives of vancomycin belonging to the class lipoglycopeptides, and teicoplanin has shown more rapid bactericidal effect than vancomycin [38]. However, if the vancomycin resistance is caused by the *vanA* operon, neither will have any antimicrobial effect through binding to the D-Ala-D-Ala motif, which is the target site for vancomycin. Teicoplanin retains activity toward VRE when the resistance is caused by the *vanB* operon [67]. Hypothetically, telavancin has a second mode of action through interactions with the bacterial membrane causing depolarization and leakage of solutes. This is independent of the D-Ala-D-Ala binding motif, which means that telavancin may be useful in treatment of infection caused by vancomycin-resistant strains [38, 58].

### Daptomycin Belongs to the Most Recently Discovered Class of Antibiotics

Daptomycin differs from other treatments in its mode of action, in that it is bactericidal, and that resistance is very rare [67, 106]. It has been reported that as many as 99.98% of *E. faecalis* and 99.82% *E. faecium* isolates are susceptible to daptomycin [106] [88], although reports on resistance range from less than 0.3% to 20% [18, 64, 107]. Daptomycin is believed to associate with the bacterial membrane, causing leakage of cellular solutes, thereby depolarization and cell death [67]. However, daptomycin susceptibility has decreased by mutations in genes such as *liaF*, *gdpD*, and *cls*, both through the occurrence of mutations in resistant strains and site-directed mutagenesis [9, 89, 118]. The mechanism of daptomycin resistance is not clear, but it is believed that the bacterial cell needs to change its membrane or trap the drug in order to divert its effects [118]. When daptomycin is administered in higher doses, there is a concern for the toxicity of the drug, as it has been found to cause increased creatine kinase levels, resulting in muscle toxicity [80].

In summary, the conventional antibiotic treatments for infections caused by VRE are often limited due to the development of resistance, as well as high dosage requirement and severe side effects including muscle and joint pain and nausea. All of these aspects make the advent of new antimicrobial therapies imperative.

### Tedizolid—a Novel Oxazolidinone

Tedizolid is a relatively new antibiotic, belonging to the oxazolidinones, like linezolid. However, tedizolid has a 4-fold lower MIC value than linezolid, and it has been shown that some strains resistant to linezolid are susceptible to tedizolid [108, 127]. Tedizolid has bacteriostatic activity against VRE and functions by targeting the 23S rRNA of the 50S subunit and impairs protein translation, much like linezolid [127]. Although clinical data is limited, it is reported that of 163 VRE strains tested, 98.8% were inhibited [108]. However, due to the similar mechanism of action to linezolid, it is likely that cross-resistance may occur, which imposes limitations in the widespread use of this antibiotic [127].

### Oritavancin—a Novel Lipoglycopeptide

Oritavancin is possibly the most promising among new antibiotic treatments of VRE infection. It currently holds approval only for treatment of acute bacterial skin and skin structure infections, ABSSI. However, it has shown promising results in animal trials of an endocarditis model [10]. Oritavancin is a semisynthetic lipoglycopeptide. Although its structure is similar to vancomycin, and it possesses the same ability to inhibit transglycosylation, oritavancin possesses an additional mechanism of action; it inhibits transpeptidation and is effective against vancomycin-resistant strains [81, 91]. It is suggested that oritavancin interacts with the bacterial membrane in a similar process as daptomycin, and causes membrane depolarization [38]. One clinical report suggested that oritavancin was successful in curing endocarditis in an elderly patient caused by *E. faecium*, although the patient experienced side effects from the treatment [62].

Although, these new treatment options are promising, it is likely that resistance mechanisms to these antibiotics will develop, especially if they are frequently used and thereby selective pressure for resistance is maintained.

## Bacteriocins Provide New Treatment Options

Development of new antibiotics is important, but there is a growing view that a new type of antimicrobials is required to stagger the ever-developing resistance. Bacteriocins are antimicrobial peptides that are produced by bacteria, often to achieve an advantage over competing bacteria in certain niches.

Most bacteria produce bacteriocins, both Gram-positive and Gram-negative species [102, 126]. There are some different classification schemes, like the 2 categories suggested by Cotter et al. [26], and they broadly agreed with Klaenhammer classification. It has also been suggested that the circular bacteriocins should be a separate class [102]. The first one consists of the lantibiotics, which are small and contain a lanthionine residue, while the second class are also small, but they do not contain a lanthionine residue. This class can be subdivided into four categories: IIa–IId. The third class consists of large heat sensitive peptides [92]. New bacteriocins are frequently discovered [126], but discovering their mechanisms of action have traditionally been more challenging [25]. However, recent advances in receptor identification via, for example, genome sequencing of resistant mutants have significantly increased the ability to elucidate bacteriocin mechanisms [25]. The knowledge of how bacteriocins exert their antibacterial effect is critical in order to further bacteriocins to in vivo treatment of infection.

Bacteriocins in general have many advantages over the traditional antibiotics. Some examples are that they may have broad or narrow spectra, target different parts of the bacterial cell than antibiotics, often have high potency and may be bioengineered because of their gene-encoded nature [27, 86, 114]. Most bacteriocins are membrane-active peptides, targeting specific components, often proteins, in target cells [27, 35, 43, 47, 85, 86]. As shown below, some of these targeted proteins play vital roles in virulence development.

Bacteriocins rarely target the same cell components as antibiotics and therefore often have potent activity against antibiotic resistant strains [27]. This has been shown in murine models where different bacteriocins have been used to treat infections caused by other resistant bacteria, for example methicillin resistant *Staphylococcus aureus*, MRSA [11, 32, 33, 121, 122]. Therefore, bacteriocins may be a potential treatment for many types of bacterial infection. However, there is a lack of new research into how bacteriocins can be used in vivo. Recently the bacteriocin AS-48 was thoroughly investigated, with positive results in a preclinical study [19]. Hence, more bacteriocins need to be put through such in depth in vivo investigations in order to promote bacteriocins to relevant treatment options.

Although the use of bacteriocins as in vivo treatment is still limited, for now, the use of bacteriocins as additives in food has been recognized for some time, especially with nisin [100, 126]. As the food industry currently requires more food preservatives with a natural origin, bacteriocins represent promising possibilities [112]. However, bacteriocins have been under-utilized and further relevant research is required.

### Combination Therapy: Bacteriocins and Antibiotics

Bacteriocins are promising treatment options alone but may be extra potent in combination treatment with synergistic antibiotics. Recently Hayes et al. published results that indicate that erythromycin and nisin have synergistic effect against strains of group B streptococcus [52]. Nisin also exhibits synergy with polymyxin B against *Acinetobacter baumannii* infections, which are nosocomial infections that are increasingly problematic [117]. Further, several combinations of nisin and antibiotics have been shown to be effective against Salmonella, both in vitro and in vivo in a murine model [113]. Chi and Holo described synergy between the bacteriocin garvicin KS and farnesol or polymyxin B against a range of bacteria, indicating that nisin is not the only bacteriocin that has synergy with the traditional antibiotics [20]. Hanchi et al. investigated synergy between durancin 61A and several traditional antibiotics, such as vancomycin and tetracycline. Durancin/vancomycin was favorably synergistic against *Staphylococcus aureus*, another critical antibiotic-resistant pathogen [51]. The synergy of antibiotics, bacteriocins, and other novel antimicrobials was described in a mini-review by Wolska et al., describing how combinatorial therapy has implications for many fields, such as the food industry, agriculture, and medicine [125]. Despite these examples, there are relatively few studies in this important area, as in other aspects of clinical bacteriocin research, that it is necessary to deal with in order to fully utilize bacteriocins and their potential.

### Enterococci as Bacteriocin Producers and Bacteriocin Targets

Enterococci are common bacteriocin producers as well as being bacteriocin targets. Some are well characterized both in their antibacterial activity and their therapeutic potential. One example of this is the bacteriocin enterocin A + B. This bacteriocin shows antibacterial activity against *Staphylococcus aureus*, *Listeria monocytogenes*, and *Escherichia coli* [6]. Another two-peptide bacteriocin that is promising against *Clostridium perfringens* is the DD14 bacteriocin, which was shown identical to bacteriocin MR10. This bacteriocin in addition did not indicate any cytotoxicity against the cell line IPEC-1, indicating that this bacteriocin may be used in vivo [17]. Very few bacteriocins have undergone significant in vivo testing, but recently Cebrián et al. described the bacteriocin AS-48, produced by different strains of enterococci. They report low haemolytic effect, lack of toxicity and pro-inflammatory effect in a murine model, taking the promise of infection treatment with bacteriocin to the next level [19]. Another example of an enterococcal bacteriocin is EntV with activity against *Candida albicans*, indicating that bacteriocin also can be used against fungal infections [49]. These bacteriocins indicate the extensive range of bacteriocins produced and their respective targets, in enterococci. This diversity clearly illustrates the untapped potential of the bacteriocins.

As described, the enterococci produce a diverse group of bacteriocins, but they are also a suitable target for other bacteriocins. Keeping in mind the issues with VRE and antibiotic resistance raised in this article, one could say that they are not only suitable targets, but that bacteriocins are necessary to combat this pathogen. There are several bacteriocins that have in vitro activity against *E. faecalis* and/or *E. faecium*, such as nisin, bacteriocin EF478, enterocin P, enterocin K1, and more [22, 78, 87, 93]; some of these will be further treated below.

### Nisin, Garvicin KS, and Bacteriocin EF478 Are Examples of Broad-Spectrum Bacteriocins

Studies have shown that nisin has been able to reduce the viability of both vancomycin-resistant *E. faecium* and *E. faecalis* in vitro, and that the supplement of nisin producing bacteria to VRE-colonized mice reduced the colonization [78]. Nisin targets the lipid II molecule of the bacterial membrane, using it as a docking molecule, and creates pores in the membrane and disrupts the proton motive force [121]. However, treating infection with nisin has not been attempted [78].

**Fig. 1:**
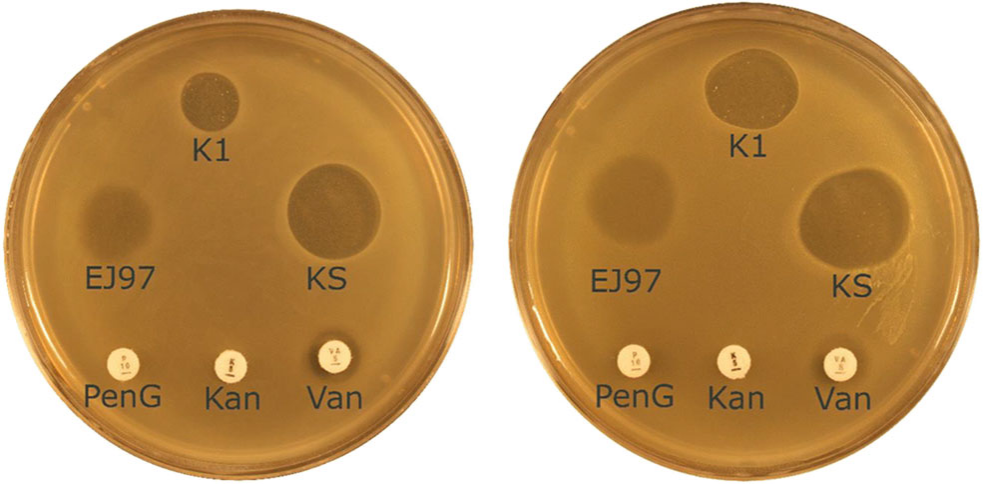
*Enterococcus faecium* is intrinsically resistant to β-lactams and aminoglycosides, represented here by penicillin G and kanamycin. In addition, they may acquire resistance to antibiotics such as glycopeptides, represented here by vancomycin. VRE is a global issue, and has the ability to cause life-threatening infection. Bacteriocins represent potential new powerful treatment modalities against antibiotic resistant bacteria. Enterocin K1, enterocin EJ97, and garvicin KS are presented in this figure. These bacteriocins show potent activity against *E. faecium* (left: LMG3593, right: LMG3104) [72]. K1 = 10 μg enterocin K1, EJ97 = 10 μg enterocin EJ97, KS = 10 μg garvicin KS, PenG = 10 μg penicillin G, Kan = 5 μg kanamycin, Van = 5 μg vancomycin

Garvicin KS is a multi-peptide bacteriocin produced by a *Lactococcus garvieae* strain isolated from contaminated raw milk [86]. It has a broad inhibitory spectrum including many Gram-positive pathogens such as MRSA and VRE, and the food-borne pathogens *Listeria* and *Bacillus* (Fig. 1). Resistance rate of *Lactococcus lactis* toward garvicin KS is quite low and the bacteriocin seems to target the PspC-mediated stress response network (unpublished data). In *E. coli*, *pspC* (also known as *ythA*) is important for the integrity and function of bacterial cell envelope [30]. In *Yersinia enterocolitica*, a *pspC* null mutant is virulent in a mouse model of infection [31]. Whether garvicin KS targets such a PspC-mediated stress response network in enterococci remains to be investigated.

Bacteriocin EF478 is a newly discovered bacteriocin showing potent activity against both *E. faecalis* and *E. faecium* [93]. Analysis indicates that this bacteriocin is a serine protease. This type of protein is known to be excreted as a toxin by other bacterial species. In addition, this bacteriocin demonstrated favorable chemical and thermodynamic qualities, indicating that it could be stable in an in vivo setting, and provide a promising new treatment option if developed [93].

### Enterocins K1 and EJ97 Have Narrow Spectrum Activity Against Enterococcal Species

We have previously shown that enterocins K1 and EJ97 are bacteriocins that have potent and relatively narrow spectrum activity against *E. faecium* and *E. faecalis*, respectively (Fig. 1)*.* They have been shown to target both vancomycin-susceptible and vancomycin-resistant nosocomial *E. faecium* and *E. faecalis* strains [87]. The bacteriocins are parts of the LsbB group of leaderless bacteriocins and have a conserved PWE motif in the C-terminus, which is important for the activity [88]. The current view of the mechanism of action is that enterocin K1 binds to the membrane-bound protein RseP, creating pores that cause leakage of solutes and other cellular constituents, thereby disrupting the proton motive force and killing the bacterial cell [87]. A structure–function relationship has also been studied for these peptides [87, 88]. They have similar structures, all with an alpha-helical motif at the N-terminal half and a non-structured part at the C-terminal half. The alpha-helical part has amphiphilic property and hence is believed to be involved in pore-forming, while the C-terminal part was demonstrated to be involved in receptor binding.

### RseP Is an Achilles’ Heel

**Fig. 2:**
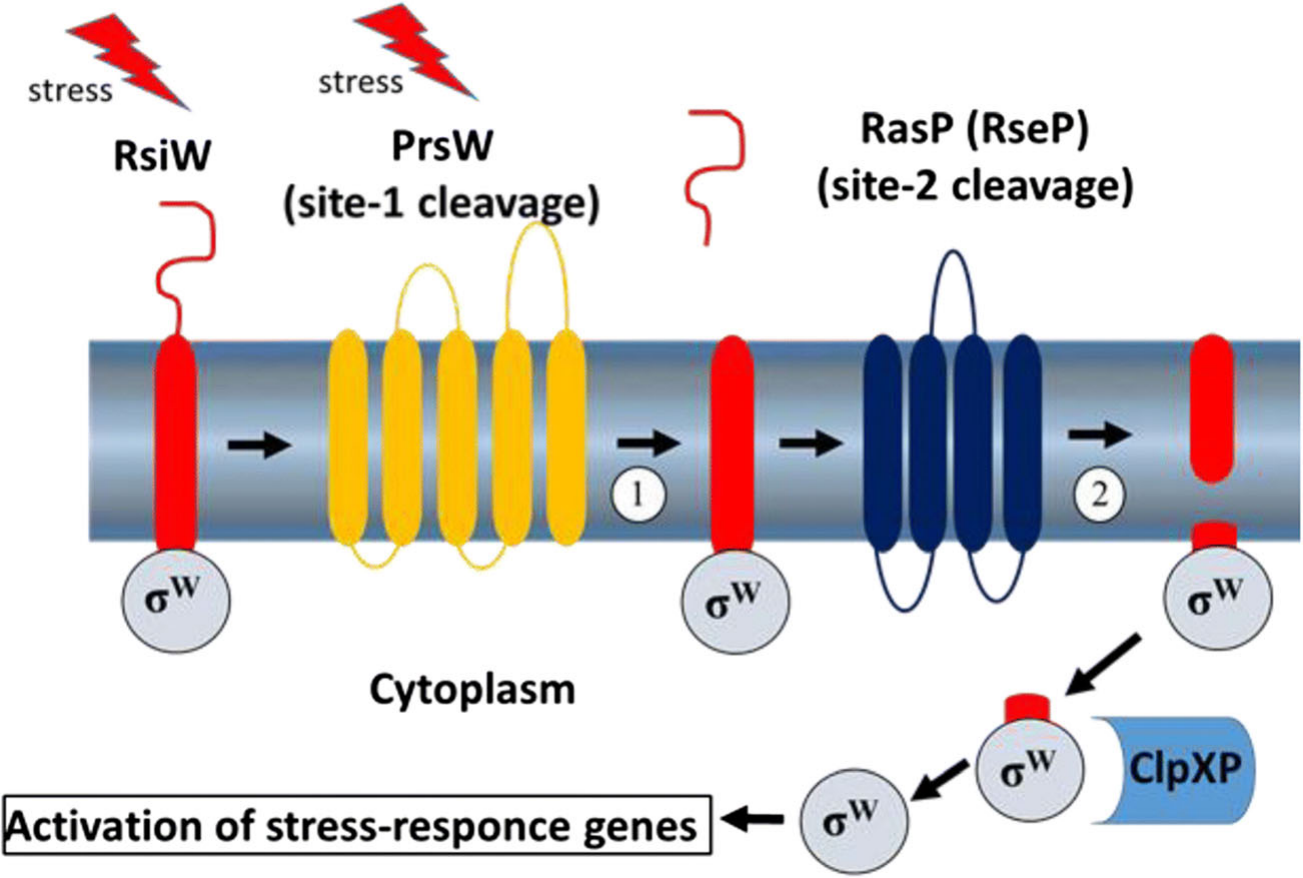
Gene activation through regulated intramembrane proteolysis [RIP] of anti-sigma factor in *B. subtilis*. Stress factors activate site-1 protease (PrsW) which cleaves anti-sigma factor RsiW at a periplasmic site followed by the second cleavage of RsiW which is carried by RasP (RseP) in the membrane. The cleaved sigma-factor undergoes further trimming by ClpXP in the cytoplasm before acting on to activate stress response genes [49]

RseP (regulator of sigma-E protease) is a membrane-bound Zn-dependent protease involved in stress response through a process called regulated intramembrane proteolysis (RIP) [14, 24]. In *E. coli*, *B. subtilis*, *E. faecalis*, and other bacteria, RseP performs the second cleavage of an anti-σ factor after cleavage by a site-1 protease [5, 44]. The release of the alternative sigma factor is crucial for bacterial response to environmental stress [99] (Fig. 2). The RseP-mediated stress response process is remarkably similar in both Gram-positive and Gram-negative bacteria [59, 63, 65].

Since RseP is crucial for the bacterial stress response in enterococci, bacteriocins targeting RseP (K1 and EJ97) not only kill sensitive bacteria but also leave resistant bacteria (with mutated RseP) to be killed by numerous environmental stressors. One such environmental stress factor is temperature. In fact, addition of K1 and EJ97 to enterococcal cells at different temperatures—comfortable (30 °C) and stressful (45 °C)—has shown that mutants appear at comfortable, but not at stressful temperatures [87]. The *rseP* gene has also been studied in vivo.

Frank et al. (2011) found that expression of *rseP* was increased during early infection, indicating that *rseP* is upregulated for infection establishment. However, deletion of *rseP* in *E. faecalis* OG1RF severely attenuated infection in an endocarditis model [46]. This indicates that the loss of *rseP* function affects the virulence of *E. faecalis*. Therefore, RseP mutants will likely not be able to establish infection, which is significant since bacteria with a functional RseP are eliminated by K1/EJ97 [87].

## Concluding Remarks and Future Perspectives

The combined effect of enterococcal intrinsic and acquired antibiotic resistance results in dangerous opportunist pathogens. The general tendency of reduced potency of existing antibiotics and very limited development of new therapeutic agents, which are often synthetic derivatives, is emphasized by increased resistance development and cross-resistance. Considering the significant economic and social burden imposed by VRE infection, it is significant to develop new treatment, as well as limit the spread of the opportunists. Bacteriocins offer new possibilities in therapy, with significant advantages that ought not to be overlooked.

Hundreds of bacteriocins have been reported in literature; however, most of them are studied as natural food preservatives or probiotics, applications which require relatively few or less strict legal regulations. This is especially the case for those produced by lactic acid bacteria which are found in diverse fermented food products and also because they are common inhabitants in our gut flora. In fact, they are often referred to as “generally regarded as safe” or GRAS. However, for medical use, bacteriocins or any new drugs are exposed to more strict regulations as they need to be carefully assessed not only for potency but also for toxicity, delivery efficacy, and other physiological and immunological parameters, in both preclinical and clinical settings. One important field in bacteriocin research which is still lagging behind is the mode of action. It is about how a bacteriocin finds the target cells at the molecular level, the following interactions between a bacteriocin and a receptor or a docking molecule, and how these interactions eventually lead to the killing of the target cells. Detailed knowledge from this field is crucial to help develop bacteriocins into drug formulations that can kill target cells efficiently, without collateral effects and development of resistant cells. We and others have identified different membrane-located proteins that are required for the sensitivity to the bacteriocins; most likely these proteins serve as receptors. The majority of them are involved in transport of sugars or amino acids across the membrane. Others are involved in stress response against antimicrobials affecting membrane integrity. We currently study the interactions between enterocins K1 and EJ97 and their receptor RseP by crystallography which hopefully will share light into their mode of action in the near future.

Bacteriocins are gene-encoded, hence their sequences can be genetically modified that may lead to new properties [61, 129]. Other modification approaches are emerging. Peptidomimetics is modification on an existing peptide that can lead to advantageous properties, such as increased stability or broadened biological activity. Interestingly, some natural bacteriocins are post-translationally glycosylated in which the glycosyl group and the target cell’s sugar transporter PST are important for the antimicrobial activity [48]. Although it has not been experimentally demonstrated yet, it is tempting to speculate the attached glycosyl group might be used as a decoy so that the entire bacteriocin can enter the target cell via the sugar-PTS, a strategy resembling the Trojan horse strategy. Future research should include such modifications on natural bacteriocins, with sugars or other chemical groups, to seek for new and favorable properties, e.g., redirecting or broadening of the target spectrum and increased diffusion.

New sources for antimicrobials to combat antibiotic resistance are now a global demand. It is no doubt that bacteriocins represent a great potential in therapeutic treatments although they are currently underexploited. With better understanding of their mode of action and new technologies to modify and increase their usefulness, bacteriocins can be the next wave of drugs or supplements for therapeutic use. Finally, but not the least important, bacteriocins are superior to antibiotics in terms of environmental-friendliness. The former are of peptides and have therefore relatively short life in nature while the latter, especially those synthetic ones, are more difficult to degrade, often leaving a long-lasting footprint in nature.
